# Analysis of patient medication compliance and quality of life of physician-pharmacist collaborative clinics for T2DM management in primary healthcare in China: A mixed-methods study

**DOI:** 10.3389/fphar.2023.1098207

**Published:** 2023-03-24

**Authors:** Jie Xiao, Qing Wang, Shenglan Tan, Lei Chen, Bingjie Tang, Shuting Huang, Yangang Zhou, Ping Xu

**Affiliations:** ^1^ Department of Pharmacy, The Second Xiangya Hospital, Central South University, Changsha, China; ^2^ Institute of Clinical Pharmacy, The Second Xiangya Hospital, Central South University, Changsha, China

**Keywords:** medication compliance, quality of life, diabetes milletus, pharmacist, primary healtcare

## Abstract

**Background:** Physician-pharmacist collaboration is a well-established care mode for the management of type 2 diabetes mellitus (T2DM) in developed countries, but no study has been conducted in primary healthcare in China. This study aims to evaluate the effects of physician-pharmacist collaborative clinics to manage T2DM in primary healthcare in China, and to better understand the factors influencing the implementation of physician-pharmacist collaborative clinics.

**Methods:** Two hundred and sixty-seven patients involved in a 12-month randomized controlled trial were assigned to physician-pharmacist collaborative clinics and usual clinics, completing surveys regarding medication compliance, quality of life (QoL) and care-seeking behavior at the baseline, 3rd, 6th, 9th and 12th month respectively, and diabetes knowledge at baseline and 12th month. A sample of twenty-two Patients, nine physicians and twelve pharmacists participated in semi-structured face-to-face interviews. The quantitative and qualitative data was integrated by triangulation.

**Results:** Patients in physician-pharmacist collaborative clinics had significant improvements in medication compliance (*p* = 0.009), QoL (*p* = 0.036) and emergency visits (*p* = 0.003) over the 12-month. Pairwise comparison showed the medication compliance score in the intervention group had been significantly improved at 3rd month (*p* = 0.001), which is more rapidly than that in the control group at 9th month (*p* = 0.030). Factors influencing the implementation of physician-pharmacist collaborative clinics were driven by five themes: pharmaceutical service, team-base care, psychological support, acceptability of care and barriers to implementation.

**Conclusion:** Integration of quantitative and qualitative findings showed the effectiveness of physician-pharmacist collaborative clinics in patient medication compliance and QoL in primary healthcare. The qualitative study uncovered barriers in insufficient clinical experience and understaffing of pharmacist. Therefore, the professional training of the primary pharmacist team should be improved in the future.

**Clinical Trial Registration:** clinicaltrials.gov, identifier ChiCTR2000031839.

## 1 Introduction

Diabetes has become a serious global health problem due to its high prevalence and associated disability and mortality (GBD, 2016 Disease and Injury Incidence and Prevalence Collaborators, 2017). Rapid economic development in the last 2 decades has led to changes in the lifestyles of the Chinese population, developing sedentary behaviors, physical inactivity and energy-dense eating habits, which have resulted in a rapidly increasing diabetes population (Pan et al., 2021; Pang et al., 2021). According to the International Diabetes Federation (IDF), the prevalence of diabetes in China is growing rapidly, ranking first in the world, at about 11.9% of the adult population (Saeedi et al., 2019). Estimates show that 116.4 million adults in China have diabetes, more than 90% of which are type 2 diabetes (T2DM), accounting for 24% of the global diabetic population (International Diabetes Federation, 2021).

Diabetes is a long-term chronic disease, and the patient’s daily behavior and self-management ability are key factors affecting the control of diabetes (Yoon et al., 2022). The management of T2DM is complex, requiring multiple interventions to optimize glucose control, reduce risk factors, and prevent complications, which is physician-dominated in China (Conlin et al., 2017; Professional Practice Committee, 2018). So far, diabetes management in China is not yet well implemented, especially in rural areas. The two main reasons for poor management are the lower density of physicians and the lower socioeconomic status and education background of patients, which are particularly common in primary healthcare (Anand et al., 2008; Wang et al., 2014; Liu et al., 2016; Jin et al., 2017). According to a national cross-sectional study in 2018, the rates of awareness, treatment and glucose control of diabetes in urban areas in China were only 40.0%, 36.2%, and 54.1%, respectively. The situation is more serious in rural areas, where rates were 32.6%, 28.8%, and 44.1% respectively (Wang et al., 2021a). There are rigorous challenges in terms of diabetes management, especially in primary medical care.

The physician-pharmacist collaborative clinics were first introduced in the United States, aiming to optimize medical treatment and reduce medical expenses (Hammad et al., 2011; Wei et al., 2022). The mode of physician-pharmacist collaboration was based on Medication Therapy Management (MTM), a service provided by pharmacists to ensure optimum therapeutic outcomes for individualization by reducing the risk of adverse events, improving patient medication compliance and quality of care (Oladapo and Rascati, 2012; Miller et al., 2016; Brandt and Cooke, 2017; Song et al., 2022). Based on comprehensive research evidence, the involvement of pharmacists in diabetes management significantly improve treatment cost-effectiveness and lower clinical parameters of glycosylated hemoglobin (HbA1c) and low-density lipoprotein (LDL) (Kiel and McCord, 2005; Farland et al., 2013).

In tertiary hospitals in China, physician-pharmacist collaborative mode has been well-established. Pharmacists conduct investigations to collect patient information, including laboratory parameters, medical and family history (Zhang et al., 2022). Pharmacists also assess medical risks and monitor adverse drug reactions to help physicians determine the therapeutic scheme. Finally, pharmacists provide medication counseling and disease education to patients (Wang et al., 2021b). However, traditional mode still dominates in primary healthcare, where physicians often make diagnoses, determine treatment plans and provide medication guidance independently, leading to several problems. As reported in previous studies, physicians were overwhelmed by the large number of patient visits, resulting in the limited time for disease education and medication instruction, and low patient satisfaction (Wen et al., 2016; Manzoor et al., 2019; Li et al., 2021).

Despite the physician-pharmacist collaborative clinics have attracted widespread attention in Chinese tertiary hospitals, no study has been carried out in Chinese primary healthcare. Therefore, we conducted a mixed-methods study to evaluate the effects and influencing factors of implementing physician-pharmacist collaborative clinics to manage T2DM in primary healthcare.

## 2 Materials and methods

**FIGURE 1:**
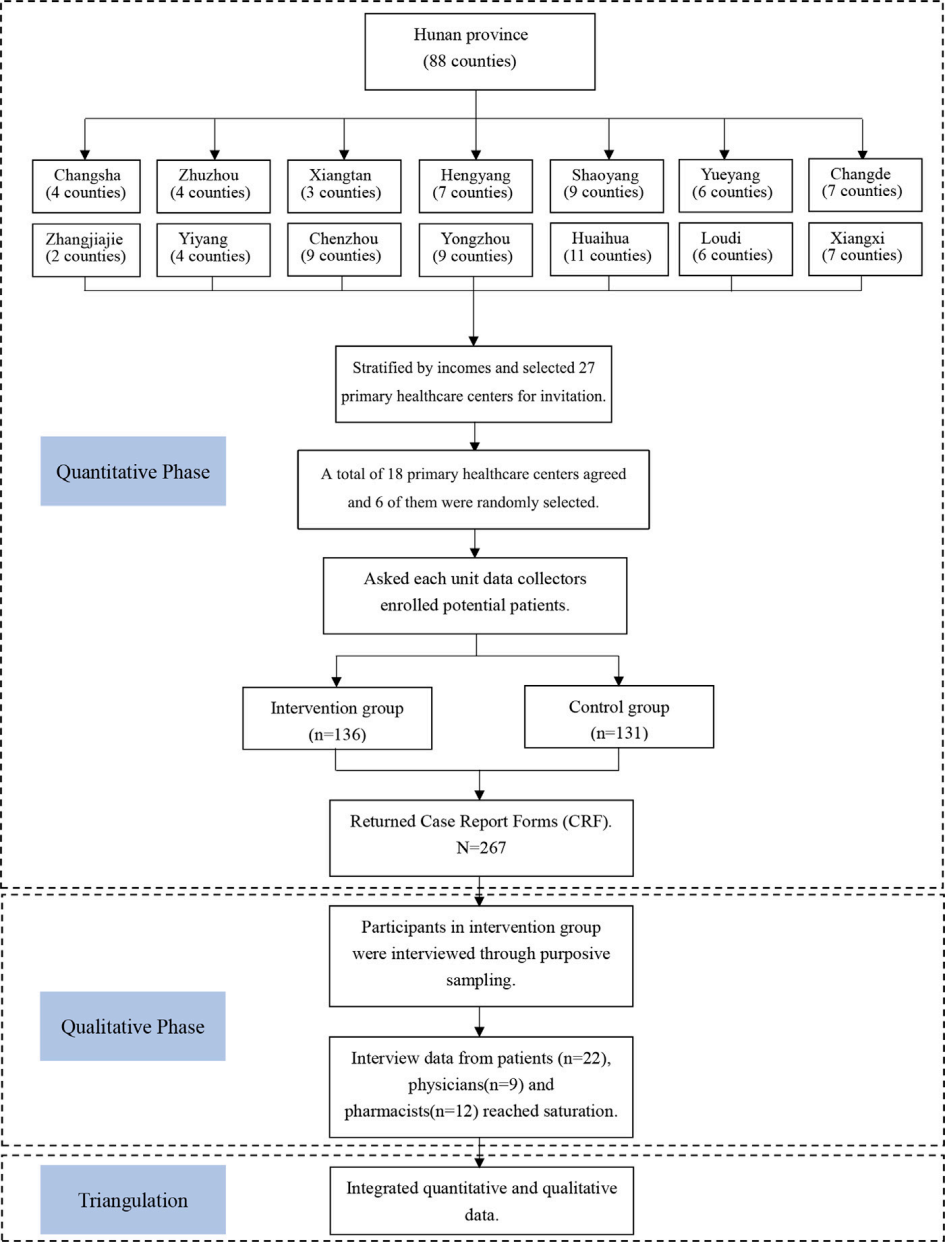
The mixed-methods study flowchart.

The mixed-methods study using an explanatory design, consists of three key components: a multi-center randomized controlled trial (RCT); and qualitative interviews with participants; and integration of quantitative and qualitative data (Figure 1). This study was reported following the Good Reporting of a Mixed Methods Study (GRAMMS) checklist and Consolidated Standards of Reporting Trials (CONSORT).

### 2.1 Research design

The study was conducted in Hunan province, which is a medium-level province in China and could represent the average medical condition in China. We randomly selected six primary healthcare centers in Hunan province (three in each group) to conduct multi-center randomized control trial. Potential patients were invited and randomly assigned to the intervention group (physician-pharmacist collaborative clinics) or the control group (usual care clinics). Patients in the intervention group underwent physician-pharmacist collaboration diabetes clinics, while patients in control group received routine clinics without pharmacist involving. A study protocol was reported in previous study (Tan et al., 2022). We collected patients’ characteristics and survey responses on medication compliance scale, EQ-5D-3L instrument and care-seeking behavior at baseline, 3rd, 6th, 9th, 12th months, and the diabetes knowledge questionnaire responses at baseline and 12th month.

Qualitative study based on descriptive phenomenological design to perceive patients’, physicians’ and pharmacists’ personal experiences, attitudes and perceptions on physician-pharmacist collaborative clinics. After the 12-month follow-ups, patients, physicians and pharmacists in the intervention group were invited to face-to-face interviews.

### 2.2 Randomization and masking

Six primary healthcare centers were selected in Hunan Province by using a standard randomization protocol (http://stattrek.com/statistics/random-number-generator.aspx). After recruitment, the six selected primary healthcare centers were randomly divided into the intervention group (physician-pharmacist collaborative clinics) or the control group (usual clinics), with three in each group. Due to the nature of pharmacist intervention, it was impossible to mask participants in the study.

### 2.3 Interventions

Local pharmacists who received standardized intervention training for 3 months at the Second Xiangya Hospital were finally assessed and qualified to participate in the collaborative clinic. Patients in the physician-pharmacist collaborative clinics received pharmaceutical care at each follow up visit, including diabetes education, medication guidance, lifestyle intervention, treatment of adverse drug reactions and identification of complications. Patients were enrolled in WeChat groups, where pharmacist conducted daily management, disease education, and send messages of diabetes knowledge, dietary advice and exercise programs. In addition, patients received peer support and encouragement from others through the WeChat group.

Both groups received routine outpatient services from physicians, including diagnosis, treatment due to limited time. There is no intervention provided by pharmacists in usual clinics.

### 2.4 Participants

Sample size is calculated using a formula for clinical trial (Billings et al., 2017). We indicated that to detect an absolute difference score of 1.14 in medication compliance in favor of the intervention group with *α* = 0.05, *β* = 0.1, and standard deviation of 1.5. Thus, a sample size of 36 patients per center was required (Tan et al., 2022). An additional 10% was added to allow for patients dropping out of the study, making a target sample size of at least 40 patients in each primary healthcare center. Participants were randomized to either intervention group or control group and performed on a 1:1 ratio. Patients were recruited in multi-center randomized control trial if: (1) have been diagnosed with T2DM; (2) 18 years or older; (3) the HbA1c level of the past 2 months was>7.5% prior to the study; (4) an informed consent signed by the patient or a legal guardian. Patients were excluded if: (1) currently pregnant; (2) have end-stage renal failure; (3) have dementia or severe psychiatric disorders; (4) receive treatment for cancer; (5) have congestive heart failure; (6) have a history of pancreatitis. The inclusion criteria for pharmacists were: (1) have received a standardized training as a clinical pharmacist; (2) have worked as a clinical pharmacist in endocrinology specialty for over 2 years; (3) be able to get comprehensive training for 3 months. Quantitative data were collected from a total of 267 patients.

Qualitative study utilized purposive sampling technique. According to maximum difference sampling, voluntarily patients, physicians and collaborating pharmacists were selected to form matching feedback models. Participants were stratified according to gender, age, educational background, course of disease and complications (for patients), working experience and professional titles (for physicians and pharmacists). Participants were recruited if: (1) took part in physician-pharmacist collaborative diabetes clinics; (2) have completed 12-month follow-ups. Recruitment continued until the researchers reached data saturation, and no participants contribute additional input in the interviews (Saunders et al., 2018). A total of 22 patients, 9 physicians, 12 pharmacists from three primary healthcare centers participated in the interviews.

### 2.5 Data collection

The primary outcomes of RCT were assessed by medication compliance scale with chronic patients, EQ-5D-3L instrument, while the second outcomes were diabetes knowledge score and care-seeking behaviour. Before the main study, we conducted a pilot study in two primary healthcare centers to evaluate the reliability of the instruments. The medication compliance scale with chronic diseases was widely used in China, with a high reliability and validity, and the Cronbach’s alpha index was 0.717 (Xu et al., 2008). The EQ-5D-3L instrument, including the visual analogue scale (VAS) and time trade-off (TTO), is widely used to assess the quality of life in China, and the Cronbach’s alpha index was 0.815 (Zhuo et al., 2018). The diabetes knowledge scale and Treatment Behaviour Survey were developed by the diabetes centre of Ruijin Hospital, Shanghai Jiao Tong University School of Medicine, which has been proved suitable for the disease control requirements of Chinese patients. The Cronbach’s alpha index of diabetes knowledge scale was 0.802 (Zhao et al., 2004).

Semi-structured face-to-face interviews were carried out by researchers from September to December 2021. To collect data in a comprehensive and detailed manner, participants were asked to provide real examples and stories of their first-hand experience in physician-pharmacist collaborative clinics. The participants were also encouraged to discuss their experiences with diabetes care. The interview outline was designed to cover the feelings of participants and their willingness to promote the implementation of physician-pharmacist collaborative clinics (Supplementary Appendix S1). The mean interview time lasted 30 min with patients, and 40 min with physicians and pharmacists, based on participants’ availability. Each interview continued until data was saturated when no more new information. All interviews were recorded with the participant’s prior permission. Audio files were transcribed by two researchers respectively to ensure data quality.

### 2.6 Data analysis

Quantitative data were analyzed using the Statistical Product and Service Solutions (SPSS). We summarized baseline characteristics using descriptive statistics and compare groups through Mann-Whitney u tests or chi-square test as appropriate. Differences in the intervention for the patient survey responses were examined with generalized estimating equations (GEE) analysis. GEE provides reliable estimate of main and interaction effects for time-dependent repeated measures data. Orthogonal polynomial contrasts were used to test for the fixed trend of mean change curve. Pairwise comparison with Dunnett’s *t*-test or Dunnett’s T3 test was also conducted to evaluate time points within each group. Missing data were not included in the analysis, as GEE approach flexibly accommodates various missing patterns and proportions (Koivusalo et al., 2016). Two-side *p*-values <0.05 were considered statistically significant.

Qualitative data were analyzed by the researchers in accordance with the Colaizzi method. The researchers read the interview transcript carefully multiple times, highlighting significant statements and assigning interpretations to them in the form of beginning codes respectively. Then the researchers identified similar viewpoints and coded them accordingly. According to the consistency of concepts, the various classes were merged, and finally, the ultimate theme concept was sublimed. Trustworthiness was achieved by credibility, transferability, dependability and confirmability (Amin et al., 2020). To ensure the credibility, purposeful sampling technique was used to select the samples with the greatest variation in participant characteristics, and participant verification was performed for the analysis and coding. Since the researchers have experience of qualitative research and interviews, they could easily communicate with the participants and extract relevant information. Researchers documented the memos and coding process in detail, and all authors from different disciplines were involved in discussions at all stages of the study to obtain the dependability criterion. Participants with different roles in collaborative clinics were interviewed to enrich perspectives, and two researchers coded separately to ensure confirmability. The perspectives of qualitative research experts and pharmacists with expertise experience were included in coding and data processing to establish confirmability. The transferability criterion was met through the depth and multi-perspective of descriptions for each category. All texts were coded by two researchers, respectively, and the contexts with inconsistent coding were discussed by all authors to reach consensus. Qualitative data were managed by NVivo 12.

### 2.7 Integration

A triangulation protocol enhances the validity and reliability of the mixed methods by exploring the convergence, complementary and dissonance (O'Cathain et al., 2010). We employed three types of triangulation techniques to integrate quantitative data and qualitative data, including investigator triangulation, methodological triangulation and data triangulation. Two researchers reviewed each data set and coded respectively, focusing on the following question: What were the factors that influenced the physician-pharmacist collaborative clinics for patient management? Researchers compared the results and data source of the two methods, and to determine whether the themes were agreement, partial agreement, silent or dissonant (Farmer et al., 2006).

### 2.8 Ethics approval

The study was approved by the Clinical Research Ethics Committee of the Second Xiangya Hospital of Central South University (No. 2019–213), and all of six primary healthcare centers accepted the ethic approval. All participants signed informed consent in the quantitative study and qualitative interview.

## 3 Results

### 3.1 Phase Ⅰ—Quantitative study

#### 3.1.1 Baseline characteristics

**FIGURE 2:**
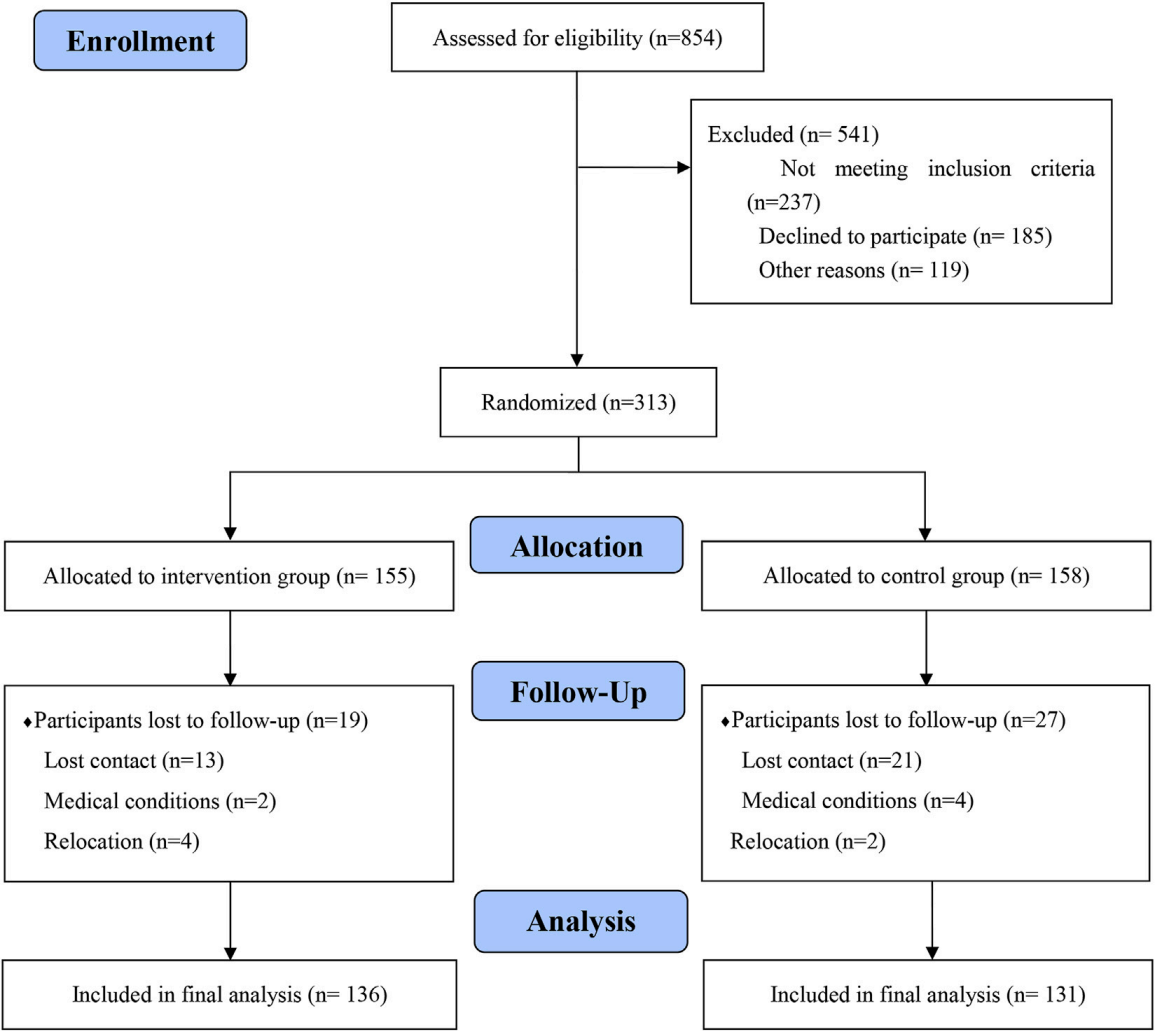
Participant recruitment and allocation.

From the total enrolled patients (*n* = 267), 50.9% (*n* = 136) participated in physician-pharmacist collaborative clinics for 12 months. The mean age of the enrolled patients was 53.7 ± 12.0 years (*n* = 267) and 56.6% (*n* = 151) are female. Overall, the mean medication compliance score was 5.57 ± 1.74. The mean TTO value was 0.972 ± 0.0587. The mean VAS index score was 70.3 ± 13.7. The diabetes knowledge score was 20.7 ± 13.7. Baseline characteristics of the two groups were generally comparable (Table 1). Figure 2 summarizes participants recruitment and allocation.

**TABLE 1:** Patients’ characteristics of quantitative samples (*n* = 267).

Characteristics	Overall (N = 267)	Intervention (n = 136)	Control (n = 131)	*p*-value
Age, years	53.7 ± 12.0	54.1 ± 9.8	53.2 ± 14.0	0.595
Gender				0.015
Male	116 (43.4%)	69 (50.7%)	47 (35.9%)	
Female	151 (56.6%)	67 (49.3%)	84 (64.1%)	
Education				0.062
Junior high school and below	169	93	76	
Senior high school	44	21	23	
College and above	54	22	32	
Income per month				0.139
<1000 RMB	65	41	24	
1000–3000 RMB	70	31	39	
>3000 RMB	132	64	68	
Occupation				0.303
Part-time job or no job	64	29	35	
Full-time job	203	107	96	
Medication compliance	5.57 ± 1.74	5.46 ± 1.62	5.68 ± 1.86	0.147
TTO	0.972 ± 0.0587	0.977 ± 0.0379	0.968 ± 0.0743	0.339
VAS	70.3 ± 13.7	70.7 ± 14.2	69.9 ± 13.2	0.421
Diabetes knowledge Scale	20.7 ± 13.7	20.6 ± 3.34	20.9 ± 4.27	0.086

#### 3.1.2 Medication compliance

**FIGURE 3:**
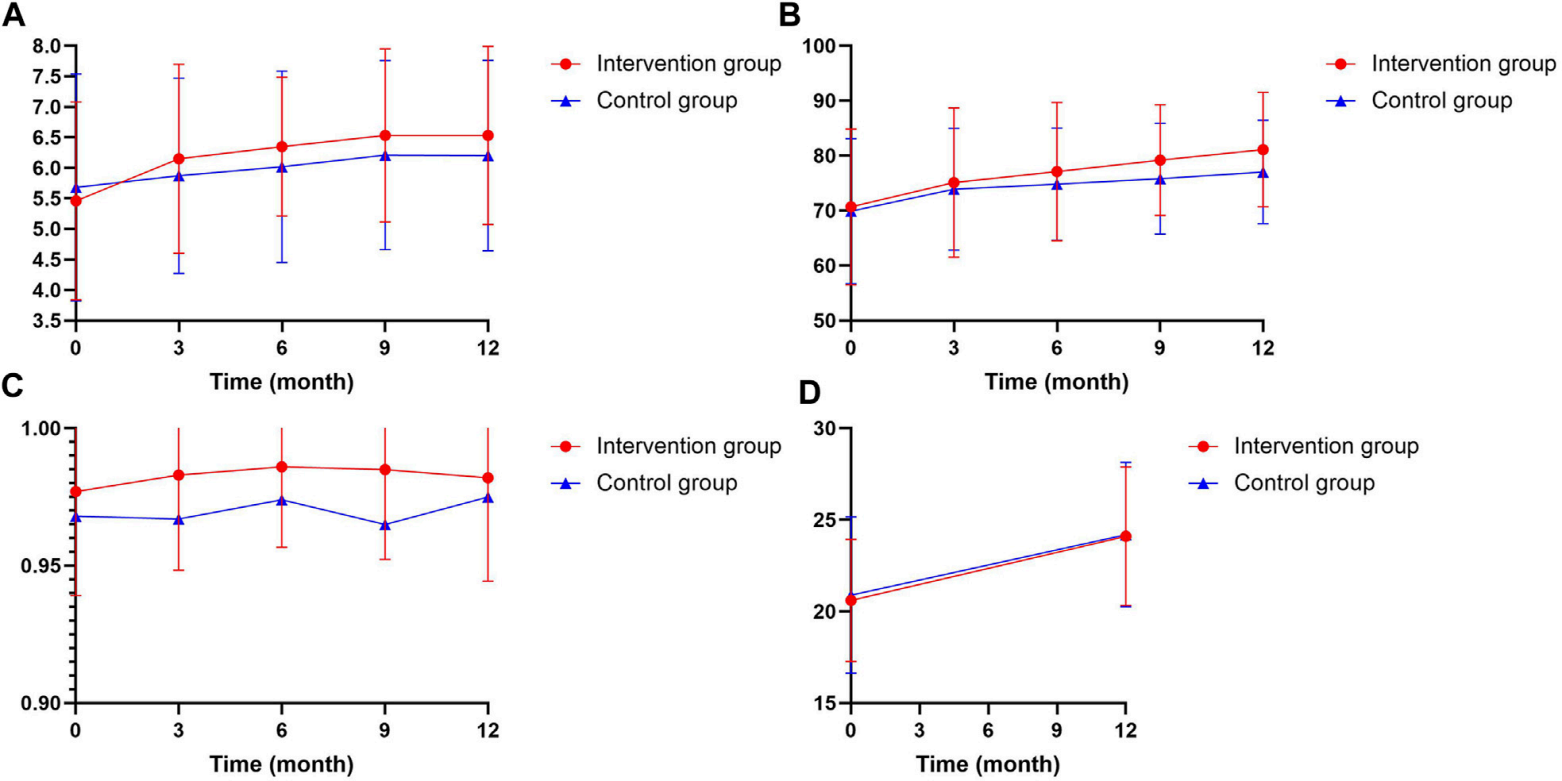
Indicated the change in medication compliance **(A)**, VAS **(B)**, TTO **(C)** and diabetes knowledge score **(D)**.

The mean improvement in medication compliance was significant between the intervention and control groups by time interaction effects over the 12 months (Collaborative clinics: 1.06 vs. Usual clinics: 0.52, *p* = 0.009). The medication compliance score in the intervention group significantly improved from 5.46 ± 1.62 at baseline to 6.15 ± 1.55 at 3 months [(CI: 0.244 to 1.13), *p* = 0.001], and improved higher to 6.53 ± 1.46 at 12 months [CI (0.620 to 1.51), *p* < 0.001] (Table 2, 3). The temporal trend curve of medication compliance in the intervention group showed a quadratic relationship (*p* = 0.004), which means the collaborative clinics significantly improved patients medication compliance in a very short period of time (Figure 3; Table 4). The medication compliance in the control group significantly improved from 5.68 ± 1.86 at baseline to 6.21 ± 1.55 at 9 months [CI [0.391 to 1.026), *p* = 0.030] and to 6.20 ± 1.56 at 12 months [CI (0.277 to 1.014), *p* = 0.035], which was 6 months later than the patients in the intervention group. The temporal trend curve showed liner trend (*p* = 0.002), indicating a gradual improvement in patients with usual care, but this process and outcomes were slower and worse than the intervention group.

**TABLE 2:** The 12-month efficacy of the physician-pharmacist collaborative clinics on medication compliance, QoL and diabetes knowledge.

Variables	The intervention group					The control group					*p*-value		
	Baseline	3 Month	6 Month	9 Month	12 Month	Baseline	3 Month	6 Month	9 Month	12 Month	Time	Group	Group × time^a^
Medication compliance	5.46 ± 1.62	6.15 ± 1.55	6.35 ± 1.14	6.53 ± 1.42	6.53 ± 1.46	5.68 ± 1.86	5.87 ± 1.60	6.02 ± 1.57	6.21 ± 1.55	6.20 ± 1.56	<0.001	0.226	0.009
TTO	0.977 ± 0.0379	0.983 ± 0.0346	0.986 ± 0.0293	0.985 ± 0.0327	0.982 ± 0.0376	0.968 ± 0.0743	0.967 ± 0.0911	0.974 ± 0.0700	0.965 ± 0.127	0.975 ± 0.0873	<0.001	0.087	0.157
VAS	70.7 ± 14.2	75.1 ± 13.6	77.1 ± 12.6	79.2 ± 10.1	81.1 ± 10.4	69.9 ± 13.2	73.9 ± 11.1	74.8 ± 10.2	75.8 ± 10.1	77.0 ± 9.43	<0.001	0.065	0.036
Diabetes knowledge Scale	20.6 ± 3.34				24.1 ± 3.78	20.9 ± 4.27				24.2 ± 3.94	<0.001	0.566	0.551

^a^ Estimated group by time interaction effects from generalized estimation equation.

**TABLE 3:** The 12-month efficacy within each group on medication compliance, QoL and diabetes knowledge.

Group	Scale		P 3rd vs. Baseline	P 6th vs. Baseline	P 9th vs. Baseline	P 12^th^vs. Baseline
Intervention	Medication compliance score		0.001	<0.001	<0.001	<0.001
	TTO		0.409	0.104	0.169	0.529
	VAS		0.015	<0.001	<0.001	<0.001
	Diabetes knowledge score					<0.001
Control	Medication compliance score		0.760	0.259	0.030	0.035
	TTO		1.000	0.946	0.995	0.940
	VAS		0.012	0.001	<0.001	<0.001
	Diabetes knowledge score					<0.001

**TABLE 4:** Time trends of medication compliance and VAS score within each group.

Group		P trend _liner_	P trend _quadratic_
Intervention	Medication compliance	<0.001	0.004
	VAS	<0.001	0.268
Control	Medication compliance	0.002	0.494
	VAS	<0.001	0.118

#### 3.1.3 Quality of life

Participants in the intervention group showed great improvement in their VAS score by time interaction effects over the 12 months compared to the usual clinics (Collaborative clinics: 10.40 vs. Usual clinics: 7.08, *p* = 0.036). But there were no significant differences by time interaction effects between groups in the TTO score (Collaborative clinics:0.00525 vs. Usual clinics: 0.00668, *p* = 0.157), although time effects were significant (*p* < 0.001).

The mean VAS score significantly improved from 70.7 ± 14.2 at baseline to 81.1 ± 10.4 at 12 months in the intervention group [(CI: 0.638 to 8.16, *p* < 0.001)], and also improved from 69.9 ± 13.2 at baseline to 77.0 ± 9.43 at 12 months in the control group [(CI: 0.680 to 7.26, *p* < 0.001)]. The temporal trend curve showed liner trend both in the intervention group (*p* < 0.001) and control group (*p* < 0.001), representing a similar VAS score growth trend, although the increments are significantly different. However, there were no significance in TTO score both in intervention group [(CI: −0.00425 to 0.0163), *p* = 0.529] and control group [(CI: −0.0288 to 0.0270, *p* = 0.940)] over the 12 months.

#### 3.1.4 Diabetes knowledge

There was no significant difference in diabetes knowledge score by time effects between the intervention group and control group over the 12 months (Collaborative clinics: 3.52 vs. Usual clinics:3.28, *p* = 0.551). The mean diabetes knowledge score for patients in the intervention group significantly improved from 20.6 ± 3.34 at baseline to 24.1 ± 3.78 at 12 months (*p* < 0.001). Similarly, the diabetes knowledge score for patients in control group also improved from 20.9 ± 4.27 at baseline to 24.2 ± 3.94 at the 12 months (*p* < 0.001).

#### 3.1.5 Care-seeking behavior

The number of care-seeking behavior showed great significance between the groups by time effects over the 12 months, including the number of outpatient visits (*p* < 0.001) and emergency visits (*p* = 0.003). Compared to control group, the number of outpatient clinic visits in the intervention group increased 1.78 times (collaborative clinics: 695 vs. usual clinics: 389), and emergency visits decreased to half (collaborative clinics: 20 vs. usual clinics: 40) in the last 12 months (Table 5).

**TABLE 5:** The 12-month care-seeking behavior of physician-pharmacist collaborative clinics.

Group	0–3 Month		3–6 Month		6–9 Month		9–12 Month		0–12 Month		P Group× time^a^	
	Outpatient	Emergency	Outpatient	Emergency	Outpatient	Emergency	Outpatient	Emergency	Outpatient	Emergency	Outpatient	Emergency
Intervention group	191	11 (8.1%)	173	3 (2.2%)	151	1 (0.73%)	180	5 (3.7%)	695	20	<0.001	0.003
Control group	94	7 (5.3%)	122	9 (6.8%)	45	8 (6.1%)	128	16 (12.2%)	389	40		

^a^ Estimated group by time interaction effects from generalized estimation equation.

### 3.2 Phase Ⅱ—Qualitative study

Five themes and seven subthemes were defined after analysis (Table 6).

**TABLE 6:** Interview themes and sub-themes with illustrative quotes.

Theme	Subtheme	Quotes from the interviews
Pharmaceutical service	rational medicine use	At present, patients are taking a large number of medicines, especially those combined with health supplements, flooding the market. (pharmacist 3)
		(We ask pharmacists) the adjustment of dosage for patients with serious complications, economical medication, and special requirements for the elderly. (physician 6)
		When my blood sugar is high, I ask my pharmacist if I need an extra dose. (patient 12)
	Medication guidance	Medication guidance of pharmacists includes instructions and adverse reactions. If the patient has other medical conditions, we will educate him about the interactions of the drugs he takes. (pharmacist 11)
		The important role of pharmacists is mainly reflected in medication instructions, as patient compliance is still a big problem, especially for patients in the remote areas in the Hunan province. (physician 7)
		I’ve also taken the drugs advertised on TV, which promotes that they could cure diabetes. Sellers of advertised drugs also text me and I’ve been scammed before. (patient 12)
	Adverse drug reactions	I get hypoglycemia sometimes, and I don't even know it. They tell me to eat something right away if I am hypoglycemic. (patient 20)
		Some of the drugs patients take have side effects. The pharmacists remind them of medication instructions, including the relief of gastrointestinal reactions after taking acarbose. (physician 4)
		Patients are encouraged to consult me instantly when they feel uncomfortable after taking medicines, such as suffering stomach pain or abdominal distension. (pharmacist 5)
Team-based Care	Education and management	Anyway, I think that is the biggest benefit that the pharmacist asks me to complete a survey of my knowledge about diabetes. They also explain to me what I did wrong. (patient 13)
		The management of diabetic patients is multifaceted, and the participation of pharmacists in patient care really achieve better outcomes. (Physician 2)
		We usually emphasize the importance of self-monitoring blood glucose to our patients. Most of them accepted us and built lasting connections with us. (pharmacist 2)
	Lifestyle intervention	I know what to eat and what not to eat. I could do anything but stop drinking. Diet and exercise are critical, and I used to taking a walk after supper. (patient 22)
		Many patients cannot maintain a balanced diet. So, I usually give them some advice, such as not eating soup, quitting smoking and drinking alcohol. (pharmacist 4)
Psychological support		When I saw the diagnosis, I cried because I didn’t know why it happened. At that time, I was very frustrated. But the doctors and pharmacists took good care of me and I am very grateful to them. (patient 8)
		In fact, the combination of drugs and a positive mindset could provide quick relief. (patient 21)
Acceptability of care		It is better to see a pharmacist and a doctor together. They are very enthusiastic and give me useful guidance. (patient 10)
		To be honest, there are so many outpatients that we probably could not do a detailed work. Pharmacists share part of the work of physicians, and even further refine our work. (physician 8)
		With our strengthened services, patients are more compliant and willing to follow up again. That makes me feel honored. (pharmacist 1)
Barriers to implementation	Insufficient clinical experience	(laugh) Giving advice on a computer is a completely different feeling than being directly in front of a clinic. If you can’t give some useful ideas and earn the trust of patients, you couldn't carry out your work. (pharmacist 1)
		There are some differences between practice and theory. It takes time to gain experience in communication skills. A comprehensive understanding of diseases and drugs is necessary as patient management is integrated. (pharmacist 2)
	Severe understaffing	I think the problem is insufficient staffing. The biggest difficulty is that pharmacists are too busy. (physician 5)
		It’s a bit difficult to fully promote the implement of collaborative clinics in primary healthcare centers, because the time and energy of pharmacists cannot meet the needs. (physician 7)
		Pharmacists should have enough time to communicate with patients. If something interferes with the communication, it could be difficult to persevere. (pharmacist 5)

### 3.3 Participant characteristics

We analyzed data from 22 patients, 9 physicians, and 12 pharmacists involved in physician-pharmacist collaborative clinics (Supplementary Appendix S2).

### 3.4 Pharmaceutical service

#### 3.4.1 Rational medicine use

During clinical treatment, pharmacists were considered experts in pharmacotherapy from patients’ and physicians’ perspectives. Many patients in primary healthcare centers tend to take more medicines when their blood glucose was high, and even take health supplements with safety risks. Additionally, polypharmacy and medication for special populations are also big challenges for physicians, in which pharmacists act as important assistants. Although the conventional medications for diabetes treatment are already familiar to physicians and the majority of patients, pharmacists also play an important role in medication safety, efficacy, economy and property in physician-pharmacist collaborative clinics.

#### 3.4.2 Medication guidance

Patients received individualized medication guidance in physician-pharmacist collaborative clinics, including administration time, drug-drug interaction, drug storage, oral and injection medication, and adverse drug reactions. Individualized medication guidance has effectively improved patients’ medication compliance, especially those combined with other diseases.

Many patients in primary healthcare centers are less educated, especially the elderly, and they are more likely to be misled by TV advertisements and false information. Due to poor knowledge of diabetes, they are easily inclined to taking fake medicines which claim to cure diabetes radically. Another pressing request from patients is to be educated to identify counterfeit medicines.

#### 3.4.3 Adverse drug reactions

Few patients experienced hypoglycemia and gastrointestinal reaction while taking hypoglycemic drugs, which reduces their medication compliance and even damages their health due to improper handling. Pharmacists teach patients to identify and handle adverse reactions in a timely manner, which really reduces emergencies. In fact, significantly fewer patients had adverse drug reactions (ADRs) in physician-pharmacist collaborative clinics due to improved medication compliance.

### 3.5 Team-based care

#### 3.5.1 Education and management

The majority of patients considered collaborative clinics have changed their misconceptions about diabetes, supporting more forms of education, such as popular medical articles, health lectures and diabetes counseling. Seven of nine physicians extremely expect more health professionals, especially pharmacists, involved in diabetes treatment and management in primary healthcare centers to make up for their lack in educating and managing patients. The participation of pharmacists in patient education and management will gradually change the *status quo* of the diabetic population. Diabetes education included disease introduction, control target and self-monitoring of blood glucose.

#### 3.5.2 Lifestyle intervention

Comprehensive management of patients with diabetes requires a balanced diet and moderate exercise. Actually, through repeated education and reminders, most of the patients controlled their diet well and engaged in physical activity, especially those overweight patients. However, despite being educated by physicians and pharmacists, 6 of 10 male patients reported smoking or drinking after illness. Supervising patients to quit smoking and alcohol will be the focus in the future physician-pharmacist collaborative clinics.

### 3.6 Psychological support

The psychological effects of suffering from diabetes were wide reaching. Nine of 22 patients reported the feeling of distress, anxiety, embarrassment and depression were continuous, resulting in psychological avoidance from treatment. Unmarried female patients described severe anxiety about getting married and having children, seeking support from physicians and pharmacists. Psychological support by medical team helps relieve the pressure of patients with diabetes, improving psychological resilience and enabling patients to have the ability to properly cope with the disease.

### 3.7 Acceptability of care

Patients who undergone treatment in diabetes described physician-pharmacist collaborative clinics as an excellent policy that was accountable for patients’ health, and they were willing to visit them in the future. Physicians explained patients in primary healthcare centers need repeated education to understand the disease and treatment properly, which required a more detailed work, and pharmacists acted as assistances of physicians in collaborative clinics. Additionally, pharmacists showed great passion for clinical practice, which allows them to leverage their expertise and motivates them to improve their professional competency.

### 3.8 Barriers to implementation

#### 3.8.1 Insufficient clinical experience

Many pharmacists described lack of clinical experience was the main obstacle in clinical procedures, of which insufficient knowledge of clinical medicine, deficient understanding of clinical need and inadequate communication skills were most frequently mentioned. Pharmacists noted that patient management is integrative and requires them to be proficient in clinical medicine, clinical pharmacy and clinical psychology, which made them spend more time on self-learning. Pharmacists who had less in-depth communications with patients reported that their professional advice lacked comprehensive consideration of patient’s clinical needs. As a result, the communication gap between pharmacist and patients appeared. Pharmacists in primary healthcare centers are aware of these problems and expect taking training courses in tertiary hospitals to enhance their professional capabilities.

#### 3.8.2 Severe understaffing

Almost all pharmacists and physicians reported the biggest barrier is the shortage of experienced pharmacists in primary healthcare centers. The limited number of trained pharmacists undertook many tasks including review of prescriptions, management of antibiotics, monitoring of adverse drug reactions and medication education for inpatients. The scarcity of pharmacists and their busy daily work hindered their participation in patient education and management and the accumulation of clinical experience, finally impeding the implementation of physician-pharmacist collaborative clinics in primary healthcare centers.

### 3.9 Phase Ⅲ—Integration

The triangulation found that pharmaceutical service and diabetes education provided by physicians and pharmacists effectively eliminated misconceptions about diabetes and hypoglycemic drugs, and improved patients’ self-management ability, resulting in a significant difference in medication compliance score between the two groups. Additionally, psychological support from physicians and pharmacists alleviated patients’ anxiety, distress and other negative emotions, manifested as a significant improvement in VAS score. In contrast, TTO score showed no improvement between the two groups, even patients in collaboratives believed it was beneficial for delaying diabetes complications.

An interesting result showed patients considered collaboratives clinics changed their misconceptions and provided them with more knowledge, but there was no significant improvement between the two groups over the 12 months. Patients high acceptance of care had led to an increase of outpatient visits. Furthermore, the high acceptance of diabetes education, including identification and management of adverse reactions, significantly reduced emergency visits.

From the qualitative data, high acceptance of participants mainly due to high-quality care, lighter burden and the enthusiasm of pharmacists, which greatly promoted the implementation of physician-pharmacist collaborative clinics. However, main barriers of insufficient clinical experience and severe understaffing of pharmacists impeded the implementation of physician-pharmacist collaborative clinics. Table 7 shows the triangulation of key qualitative and quantitative findings.

**TABLE 7:** Triangulation outcomes from integrating quantitative and qualitative data.

Quantitative data	Qualitative data	Triangulation outcome
Patients in collaborative clinics got improved medication compliance score more effectively and rapidly than usual care	Patients in collaborative clinics perceived the pharmaceutical service and diabetes education as effective measures	Agreement
Patients in collaborative clinics received higher VAS scores than usual care, but the same TTO scores	Psychological support alleviated patients’ anxiety, distress, embarrassment and depression. Most patients believed collaborative clinics help delay the diabetes complications	Partial agreement
Diabetes knowledge score improved in both two groups over time, but there was no difference between the groups	Patients considered collaborative clinics changed their misconceptions about diabetes due to the implementation of pharmaceutical service and team-based care	Dissonance
Patients in collaborative clinics showed a higher number of outpatient visits and a lower number of emergency visits	Patients expressed high acceptance and recognition of collaborative clinics. Patients in collaborative clinics learnt to identify and handle adverse reactions	Agreement
No quantitative data were collected to identify enablers	Patients described collaborative clinics as a good policy. Physicians thought collaborative clinics with pharmacists did reduce their burden. Pharmacists showed great enthusiasm for clinics practice	Silence
No quantitative data were collected to identify barriers	Main barriers for implementation of physician-pharmacist collaborative clinics were insufficient clinical experience and severe understaffing of pharmacists	Silence

## 4 Discussion

This study integrated findings from the quantitative and qualitative data, providing a conducive perspective through which to evaluate the effects of physician-pharmacist collaborative clinics. In the 12-month RCT among patients with diabetes, we found that patients in collaborative clinics improved medication compliance and QoL more effectively and rapidly than usual care. As a result, the number of outpatient visits for patients in collaborative clinics increased while the emergency visits significantly decreased. The four thematic domains from qualitative study showed that pharmaceutical service, team-base care, psychological support and acceptability of care were the core of physician-pharmacist collaborative clinics, providing better healthcare for diabetic patients in primary healthcare. These findings demonstrate that the physician-pharmacist collaborative clinics effectively achieve better patient outcomes by improving medication compliance and QoL.

The integration of quantitative data and qualitative data showed partial agreement in the difference of QoL and dissonance in diabetes knowledge score between groups. It is worth noting that the TTO score at baseline both in the intervention group (0.977 ± 0.0380) and the control group (0.968 ± 0.0743) are better than the average of 2017 Chinese Heilongjiang population TTO score (0.959) (Huang et al., 2017). A mainly reason for this may be that most of patients included the trail had no complications that affect daily life. Due to the long process of the emergence of diabetic complications, the main impact of diabetes on early stage patients was reflected in the VAS score.

However, we noticed that both groups showed improvements in the medication compliance and VAS scores, despite the collaborative clinics being more effective and rapid. It means that the routine treatment in primary healthcare also has a positive effect on patients, but takes longer and carries a greater risk of complications. We also found that diabetes knowledge score showed great improvement in both two groups over the 12 months, indicating no additional benefit from the collaborative clinics. In fact, a survey on the awareness rate of diabetes knowledge in Chinese counties reported that diabetes-related knowledge of patients with T2DM was mainly acquired through media such as television or the Internet, accounting for 85.58%, followed by books or newspapers, accounting for 32.84% (Shao et al., 2022). This suggests that the traditional education mode seems to be ineffective in primary healthcare, and that the education mode in collaborative clinics needs to be combined with common and fast-spreading media.

We compared the sociodemographic characteristics of the patients to illustrate comparability between the two groups. There was a difference in gender between the two groups at baseline. As a matter of fact, the dramatic increase in T2DM and related complications is accompanied by evidence of clinical gender differences, in which both biological and psychosocial factors contribute to gender differences in diabetes risk and outcome (Kautzky-Willer et al., 2016). Overall, psychosocial stress appears to affect women more than men. However, previous studies have showed that gender differences have no influence on medication compliance and diabetes knowledge score, but educational background had a greater influence (Zhao et al., 2004). Therefore, gender differences between the two groups at baseline did not affect the results of this study.

Effectiveness of physician-pharmacist collaborative clinics for T2DM management has been proved by many clinical studies. By providing comprehensive medication education and patient management, pharmacists helped patients reduce HbA1c, LDL cholesterol, total cholesterol and blood pressure, further reducing emergency events compared to usual care (Shane-McWhorter and Oderda, 2005; Aguiar et al., 2018; Matzke et al., 2018). Moreover, pharmacists’ qualifications and service contribute to better healthcare and a favorable return on investment of $1.1–2.8 million dollars for patients (Lehn et al., 2019). Pharmaceutical care, pharmacist counselling and diabetes management provided by pharmacists have been proved to improve patient outcomes (Shao et al., 2017; Lum et al., 2019; Besemah et al., 2021).

A burning question over diabetes healthcare in primary healthcare of China is to maintain stable glucose control, delay diabetic complications and reduce emergency events, which require an effective and feasible healthcare mode. The diabetes population in primary healthcare, especially in rural China, has been increasing dramatically due to multiple reasons, including the growth of the economy, the aged tendency of the population, and the changed lifestyle (Huang et al., 2022). Diabetes management, as the most important part of treatment, includes physician’s management and patient self-management. However, both two aspects are insufficient in primary healthcare. Firstly, the number of physicians in China ranks forefront in the world with 2.27 per 1000, slightly less than the United State with 2.61 per 1000 in 2019 (World Health Organization, 2022). However, there is a serious maldistribution of physician density, which in urban areas was more than twice that in rural areas (Anand et al., 2008). Moreover, each physician per 1,000 population added to primary healthcare centers was associated with 67% more outpatient visits (Anand et al., 2008). However, certified physicians tend to serve in provincial urban centers rather than in rural areas due to multiple reasons, including low income, limitations in their qualification and career development (Hu et al., 2017; Wang et al., 2021c). Therefore, it’s necessary to ensure and improve the quality and quantity of health professionals in primary healthcare, especially in rural areas. Secondly, the lower socioeconomic status and educational background of rural patients limit their diabetes self-management (Wallace et al., 2010; Le et al., 2016). Studies show that patients with a higher level of education had a greater probability of regularly self-monitoring blood glucose, medication compliance, and taking measures to control diabetes (Hill et al., 2013).

Actually, with the development of clinical pharmacy, pharmacists have gradually completed the transition from pharmacy to clinical practice. They made a lot of attempts in the process, including pharmacotherapy clinics (Shahrami et al., 2022), MTM service (Johnson et al., 2018), and patient centered pharmacist care (Paneerselvam et al., 2022). Those pharmacists with rich pharmacotherapy knowledge reserves, as irreplaceable partners of physicians, ensure rational drug use, improve patient medication compliance, and ultimately ameliorate patient outcomes (Marr et al., 2018). Under the brunt of Corona Virus Disease 2019 (COVID-19), pharmacists motivated by medical professionalism were reassigned to new tasks, such as disseminating educational materials, intensive care and remote clinical service (Hao et al., 2021). Undoubtedly, pharmacists play a more important role in healthcare during the special time.

Due to the COVID-19 pandemic, medical resources have been reallocated, resulting in reduced outpatient clinics, which inevitably affected chronic disease management. Recently, research focused on diabetes management has shifted from outpatient clinics to online remote management. However, trials with larger populations tend to be negative, especially among patients broadly recruited from primary healthcare (Jia et al., 2021). One obvious reason is that eHealth platforms seem not user-friendly for patients, particularly for elderly users who are the major group of diabetics (Gao et al., 2017). Moreover, remote training may spend longer time for patients, resulting in poor patient adherence (Heselmans et al., 2020). Conversely, pharmacists in collaborative clinics play an important role in diabetes education, management, follow-up visits and remote introduction if necessary during the COVID-19 pandemic.

However, the main barriers to implement physician-pharmacist collaborative clinics are the lack of clinical experience and understaffing of pharmacists. In fact, the education background and knowledge structure of pharmacists in primary healthcare are far from the current clinical needs, resulting in their poor capability in guiding clinical practice, especially for the highly educated population (Hu et al., 2014). Overall, it’s more important to carry out professional training for pharmacists in primary healthcare, including knowledge in disease, communication skills and comprehensive management capabilities (Huang et al., 2021). Due to the long payback period, many primary healthcare centers do not attach importance to the development of pharmacy departments. Fortunately, pharmacists participate in clinical practices gradually, which is conducive to the development of pharmacy and the transformation of pharmacists in primary healthcare. In our enrolled intervention healthcare centers, Taoyuan People’s Hospital was the first to officially launch physician-pharmacist collaborative clinics for T2DM management.

This study has several limitations. First, unobserved confounding bias cannot be ruled out in this study. Second, the sample size was calculated based on primary outcomes, that may restrict the representative of this study. All primary healthcare centers are from Hunan Province, which may not reflect the situation in other regions of China. Third, this study could not be blinded, and Hawthorne effect could appear.

This study is the first mixed study to explore the effects and influencing factors of implementation of physician-pharmacist collaborative clinics in primary healthcare in China. Implementation of physician-pharmacist collaborative clinics had effectively strengthened patient management, especially the management of patient medication compliance and QoL. Through the pharmaceutical service, team-based care, psychological support and acceptability of care, physician-pharmacist collaborative clinics improved patient medication compliance and QoL more effectively and rapidly than usual care, which had been highly commended by patients. Our findings suggest that the insufficient clinical knowledge and severe understaffing of pharmacists should be considered in primary healthcare. In the future, professional training of primary pharmacist should become the focus of pharmacy practice to enhance patient-centered care in primary healthcare. Our findings provide evidence of the effectiveness of physician-pharmacist collaborative clinics and lay a solid foundation for further development and implementation of collaborative clinics in primary healthcare.

## Data Availability

The raw data supporting the conclusion of this article will be made available by the authors, without undue reservation.
