# Supplementary material for: Analysis of patient medication compliance and quality of life of physician-pharmacist collaborative clinics for T2DM management in primary healthcare in China: A mixed-methods study
Source: Front Pharmacol. 2023 Mar 24;14:1098207. doi: 10.3389/fphar.2023.1098207 (PMC10080104; doi:10.3389/fphar.2023.1098207)
Supplement: Supplementary file 1 [file Table1.DOCX]

**Supplementary appendix 1 Interview guide**

**1 Interview guide in patient**

**1.1 Patient experiences of participating in Physician-Pharmacist collaborative clinics**

**Q1: Have you ever been to the Physician-Pharmacist collaborative clinics before our clinical trial?**

- What kind of Physician-Pharmacist collaborative clinics did you attend?

**Q2: Why did you participate in Physician-Pharmacist collaborative clinics?**

- What do you expect from the Physician-Pharmacist collaborative clinics?
- Has your expectation been fulfilled?

**Q3: How do you feel about the Physician-Pharmacist collaborative clinics?**

- What is the difference between the Physician-Pharmacist collaborative clinics and the usual clinic you visited before?
- Do you think visits in Physician-Pharmacist collaborative clinic are helpful for your diabetes treatment? Can you give me an example?

**Q4: What services does the pharmacist provide in** **the Physician-Pharmacist collaborative clinics? Can you describe in detail?**

- What do you think of the services?
- Which services are great? What other services do you need?

**Q5: How would you rate pharmacists’ work in Physician-Pharmacist collaborative clinics?**

- Are pharmacists up to the job?

**Q6: Do you feel comfortable communicating with pharmacists?**

- Are you willing to accept the pharmacist's advices and strictly implement them?
- Which advice have not been implemented? Can you tell me the reasons?

**Q7: What is the impact of Physician-Pharmacist collaborative clinics on patients compared to usual clinics?**

- What conveniences and inconveniences does the Physician-Pharmacist collaborative clinics bring to you? Can you tell me the reasons?

**1.2 Patient willingness to promote the implementation of** **Physician-Pharmacist collaborative clinics**

**Q8: After the follow-ups of our project, if the hospital conducts a Physician-Pharmacist collaborative clinic, are you willing to choose?**

- Are you willing to introduce other diabetic patients to the Physician-Pharmacist collaborative clinics?

**Q9: What other diseases can be treated by Physician-Pharmacist collaborative clinics?**

**Q10: What advice do you have for Physician-Pharmacist collaborative clinics?**

**2 Interview guide in physician**

**2.1 Physician experiences of participating in** **Physician-Pharmacist collaborative clinics**

**Q1: Will you introduce** **treatment schedule to the patient when prescribing in usual clinics?**

- How are your patients performing in your treatment schedules? /Are they executing your treatment schedules properly?
- How are the patients’ compliance?
- What is the difference between patients participating in Physician-Pharmacist collaborative clinics and usual clinics?

**Q2: How would you rate pharmacists’ work in** **Physician-Pharmacist collaborative clinics?**

- When will you communicate with pharmacists/ask the pharmacists’ opinion?
- Would you take advices of pharmacists? Can you give me an example?

**Q3: Do you feel comfortable communicating with pharmacists?**

- What problems did you encounter in your communication with the pharmacists?

**Q4: What do you think of the** **pharmaceutical services provided by the pharmacists in Physician-Pharmacist collaborative clinics?**

- What does pharmacists do in Physician-Pharmacist collaborative clinics?
- What are the advantages and disadvantages of Physician-Pharmacist collaborative clinics?
- What kind of work do pharmacists need to do? /What advice do you have for pharmacists’ work?
- If pharmacists want to provide pharmaceutical services, which way is the most direct and effective?
- How has your perception of pharmacists changed through Physician-Pharmacist collaborative clinics?

**Q5: What is the impact of Physician-Pharmacist collaborative clinics on physicians compared to usual clinics?**

- What conveniences and inconveniences does the Physician-Pharmacist collaborative clinics bring to you? Can you tell me the reasons?

**Q6: What is the impact of Physician-Pharmacist collaborative clinics on patients compared to usual clinics?**

**2.2 Physician willingness to promote the implementation of Physician-Pharmacist collaborative clinics**

**Q7: After the follow-ups of our project, if the hospital conducts a Physician-Pharmacist collaborative clinic, are you willing to participate in?**

**Q8: What other diseases can be treated by Physician-Pharmacist collaborative clinics?**

**Q9: What advice do you have for Physician-Pharmacist collaborative clinics?**

**Q10: What are the enablers and barriers in promoting the implementation of Physician-Pharmacist collaborative clinics in primary hospitals?**

**3 Interview guide in pharmacist**

**3.1 Pharmacist experiences of participating in Physician-Pharmacist collaborative clinics**

**Q1: What are your main responsibilities as a clinical pharmacist/pharmacist?**

- Have you ever known Physician-Pharmacist collaborative clinics? / What are the differences between what you learned and what you experienced?
- What kind of work do you mainly undertake in Physician-Pharmacist collaborative clinics?

**Q2: Do you think the communication with physicians is smooth and comfortable?**

- What problems did you encounter in your communication with the physicians?
- When did you communicate with physicians?
- Would you give advises to physicians? Did they accept? Can you give an example?

**Q3: Do you feel comfortable communicating with patients?**

- Did the patients accept your advice and implement it accurately? Can you give an example?
- How were the patients’ compliance?

**Q4: How has the Physician-Pharmacist collaborative clinics affected pharmacists?**

- How would you rate your work?

**Q5: What is the impact of Physician-Pharmacist collaborative clinics on pharmacists compared to usual clinics?**

- How will you improve your work?
- Do you have other ways to provide pharmaceutical services?

**3.2 Pharmacist willingness to promote the implementation of Physician-Pharmacist collaborative clinics**

**Q6: Did any patient come to you for consultation in outpatient clinic?**

- What did patients ask you about?

**Q7: After the follow-ups of our project, if the hospital conducts a Physician-Pharmacist collaborative clinic, are you willing to participate in?**

**Q8: What other diseases can be treated by Physician-Pharmacist collaborative clinics?**

**Q9: What advice do you have for Physician-Pharmacist collaborative clinics?**

**Q10: What are the enablers and barriers in promoting the implementation of Physician-Pharmacist collaborative clinics in primary hospitals?**

**Q11: What would pharmacists do to promote the implementation of Physician-Pharmacist collaborative clinics?**
