# Supplementary material for: Analysis of patient medication compliance and quality of life of physician-pharmacist collaborative clinics for T2DM management in primary healthcare in China: A mixed-methods study
Source: Front Pharmacol. 2023 Mar 24;14:1098207. doi: 10.3389/fphar.2023.1098207 (PMC10080104; doi:10.3389/fphar.2023.1098207)
Supplement: Supplementary file 2 [file Table2.DOCX]

**Supplementary appendix 2 Baseline characteristics of qualitative samples**

**Table S1. Patients’ characteristics of qualitative samples (n=22)**

| Characteristics | Number | Percentage |
| --- | --- | --- |
| Gender |  |  |
| Male | 10 | 45% |
| Female | 12 | 55% |
| Age |  |  |
| ≤30 | 1 | 5% |
| 31-50 | 5 | 22% |
| 51-70 | 15 | 68% |
| 70+ | 1 | 5% |
| Education |  |  |
| Primary school | 4 | 18% |
| Middle school | 10 | 46% |
| High school | 4 | 18% |
| College or above | 4 | 18% |
| Employment Status |  |  |
| Ⅰ Non-manual workers | 3 | 14% |
| Ⅱ Few-manual workers | 6 | 27% |
| Ⅲ manual workers | 13 | 59% |
| Agriculture & animal husbandry | 9 | 41% |
| Construction industry | 2 | 9% |
| Factory worker | 2 | 9% |
| Years in disease course |  |  |
| <5 | 11 | 50% |
| 5-10 | 4 | 18% |
| 11-15 | 4 | 18% |
| >15 | 3 | 14% |

**Table S2. Physicians’ characteristics of qualitative samples (n=9)**

| Characteristics | Number | Percentage |
| --- | --- | --- |
| Gender |  |  |
| Male | 4 | 44% |
| Female | 5 | 56% |
| Age |  |  |
| ≤40 | 4 | 45% |
| 41-50 | 3 | 33% |
| 50+ | 2 | 22% |
| Highest academic qualification |  |  |
| Bachelor degree | 6 | 66% |
| Postgraduate degree | 2 | 23% |
| Doctoral degree | 1 | 11% |
| Professional title |  |  |
| Attending physician | 3 | 33% |
| Deputy director | 3 | 33% |
| Director | 3 | 33% |
| Years in endocrinology |  |  |
| <10 | 1 | 11% |
| 10-20 | 3 | 33% |
| 20-30 | 4 | 45% |
| 30+ | 1 | 11% |

**Table S3. Pharmacists’ characteristics of qualitative samples (n=12)**

| Characteristics | Number | Percentage |
| --- | --- | --- |
| Gender |  |  |
| Male | 3 | 25% |
| Female | 9 | 75% |
| Age |  |  |
| ≤40 | 8 | 67% |
| 41-50 | 3 | 25% |
| 50+ | 1 | 8% |
| Highest academic qualification |  |  |
| Bachelor degree | 7 | 58% |
| Postgraduate degree | 5 | 42% |
| Professional title |  |  |
| Pharmacist | 2 | 17% |
| Attending pharmacist | 4 | 33% |
| Deputy director | 5 | 42% |
| Director | 1 | 8% |
| Years in pharmacy |  |  |
| <10 | 3 | 25% |
| 10-20 | 5 | 42% |
| 20-30 | 3 | 25% |
| 30+ | 1 | 8% |
| Years in clinical pharmacy |  |  |
| <5 | 5 | 42% |
| 5-10 | 4 | 33% |
| 10+ | 3 | 25% |
